# CARING FOR INDIVIDUALS WITH DEMENTIA AND COGNITIVE IMPAIRMENT IN LOS ANGELES: A CAREGIVER PROFILE

**DOI:** 10.1093/geroni/igac059.2049

**Published:** 2022-12-20

**Authors:** Ariane Thomas, Noel Barragan, Mariana Reyes, Tony Kuo

**Affiliations:** Los Angeles County Department of Public Health, Los Angeles, California, United States; Los Angeles County Department of Public Health, Los Angeles, California, United States; Los Angeles County Department of Public Health, Los Angeles, California, United States; University of California, Los Angeles, Los Angeles, California, United States

## Abstract

In 2020, the Los Angeles County Department of Public Health launched Healthy Brain LA (HBLA). HBLA is a multi-pronged project designed to promote cognitive health, reduce the risk of dementia, and help develop a dementia-focused strategic plan for Los Angeles County (LAC) that prioritizes the needs of those impacted by dementia and cognitive impairment and their caregivers. To support this effort, the HBLA team analyzed weighted data from the 2019 and 2020 California Health Interview Survey (CHIS) to better understand the profile of adult caregivers of individuals with dementia and cognitive impairment. CHIS is a publicly available, population-based, web and telephone survey that asks California residents about a wide range of health topics, yielding representative data on all 58 counties in the state. In 2019-2020, an estimated 1.6 million adults provided care to individuals aged 18 years or older in LAC. About 322,880 of those caregivers provided care for individuals with dementia and cognitive impairment, representing 2% of LAC’s adult population. Many of these caregivers were female (59%), Latino/Hispanic (44%), and between 50-64 years of age (32%). Most of them experienced challenges, such as financial stress (61%), physical/mental health problems (18%), or a change in job status (27%), due to caregiving. These data provide the first local estimate of dementia and cognitive impairment caregiving in nearly 15 years. Results offer critical information about this population that will be used by the HBLA team and its partners to guide efforts to effectively meet the needs of caregivers in LAC.

